# Soil Aggregates and Associated Organic Matter under Conventional Tillage, No-Tillage, and Forest Succession after Three Decades

**DOI:** 10.1371/journal.pone.0084988

**Published:** 2014-01-20

**Authors:** Scott Devine, Daniel Markewitz, Paul Hendrix, David Coleman

**Affiliations:** 1 Warnell School of Forestry and Natural Resources, The University of Georgia, Athens, Georgia, United States of America; 2 Odum School of Ecology, The University of Georgia, Athens, Georgia, United States of America; University of British Columbia, Canada

## Abstract

Impacts of land use on soil organic C (SOC) are of interest relative to SOC sequestration and soil sustainability. The role of aggregate stability in SOC storage under contrasting land uses has been of particular interest relative to conventional tillage (CT) and no-till (NT) agriculture. This study compares soil structure and SOC fractions at the 30-yr-old Horseshoe Bend Agroecosystem Experiment (HSB). This research is unique in comparing NT and CT with adjacent land concurrently undergoing forest succession (FS) and in sampling to depths (15–28 cm) previously not studied at HSB. A soil moving experiment (SME) was also undertaken to monitor 1-yr changes in SOC and aggregation. After 30 years, enhanced aggregate stability under NT compared to CT was limited to a depth of 5 cm, while enhanced aggregate stability under FS compared to CT occurred to a depth of 28 cm and FS exceeded NT from 5–28 cm. Increases in SOC concentrations generally followed the increases in stability, except that no differences in SOC concentration were observed from 15–28 cm despite greater aggregate stability. Land use differences in SOC were explained equally by differences in particulate organic carbon (POC) and in silt-clay associated fine C. Enhanced structural stability of the SME soil was observed under FS and was linked to an increase of 1 Mg SOC ha^−1^ in 0–5 cm, of which 90% could be attributed to a POC increase. The crushing of macroaggregates in the SME soil also induced a 10% reduction in SOC over 1 yr that occurred under all three land uses from 5–15 cm. The majority of this loss was in the fine C fraction. NT and FS ecosystems had greater aggregation and carbon storage at the soil surface but only FS increased aggregation below the surface, although in the absence of increased carbon storage.

## Introduction

Understanding the mechanisms for soil carbon storage and stabilization relative to land management is increasingly relevant as soil organic carbon (SOC) is known to be a major pool of global C and SOC is critical to sustaining soil productivity. Since physical protection of SOC within stable soil aggregates is considered to be one of the major SOC stabilization mechanisms, [1]–[3], the effect of land management on aggregate stability is accepted as a key factor in determining SOC levels [4]. There have been many comparisons of soil aggregates under conventional tillage relative to no-tillage agriculture but relatively few that have incorporated other land uses such as abandoned agricultural fields or forest succession, particularly over a decades timescale.

The three-decade agroecosystem experiment at Horseshoe Bend (HSB), Athens, GA, USA, has long focused on SOC differences in side-by-side plots of conventional tillage agriculture (CT) and no-till agriculture (NT). This research has demonstrated increased SOC in the 0–5 cm layer of NT [5] and some role for aggregation in SOC protection [6]. More recent work at HSB has incorporated adjacent plots of forest succession (FS), which has demonstrated a similar increase in 0–5 cm SOC relative to CT as well as an increase in particulate organic carbon (POC) in 5–15 and 15–30 cm subsurface soil layers [7]. Although previous CT-NT comparisons have been reported from HSB this study extends analyses to subsurface soil layers (15–28 cm) previously not studied at HSB for aggregation. These subsurface soils are recognized to affect the carbon balance of CT-NT studies [8]. Furthermore, few studies have compared CT and NT with other land uses but here we simultaneously investigate the role of aggregation in forest succession to evaluate the robust nature of aggregates for SOC stabilization. As such there is an opportunity to understand if physical stabilization through aggregation is functioning similarly in 0–5 cm soils in NT and FS, and if aggregation in subsurface layers of FS is stabilizing POC at rates greater than under agroecosystems.

Under these contrasting land uses (CT-NT-FS) soil structure can both directly and indirectly affect SOC stabilization. Narrow pores can directly exclude extracellular enzymes, microbes, and soil micro-fauna. Pore size also indirectly affects decomposition by regulating fluxes that influence microbial activity, such as oxygen, heat, solutes, and water. This explains how intra-aggregate pores, which are finer (and perhaps more tortuous) than inter-aggregate pores [9], can slow decomposition within aggregates. The abundant research on the relationship between soil aggregation and SOC has produced several contrasting models of how physical protection of SOC occurs.

The aggregate hierarchy model proposes that soil structure arises from aggregation of fine mineral particles into microaggregates (53 µm to 250 µm) continuing to aggregate to macroaggregates (>250 µm) with increasing dependence on more transient, organic binding agents as the scale increases [10]. This model suggests that SOC concentration increases with increasing aggregate size and that cultivation exposes these binding agents to microbial attack with subsequent loss of SOC [11].

Oades [12] later modified this theory hypothesizing that microaggregates formed around POC (53–2000 µm) when enmeshed within stable macroaggregates. Thus, soils lacking mechanical disturbance such as NT or FS result in decreased macroaggregate turnover and higher SOC due to microaggregate formation and stabilization of fine POC (53–250 µm) [13], [14]. Loss of SOC in cultivated soils is explained by shorter macroaggregate turnover times and a reduced opportunity for formation of microaggregates around a fine POC core [3].

More recently, it has been hypothesized that increased pore connectivity in soils that are not tilled allows greater movement of dissolved organic carbon (DOC) from larger pores surrounding aggregates into intra-aggregate pore spaces [15]. In tilled soils, disruption of the pore network effectively isolates substantial portions of the intra-aggregate pore space from replenishment of DOC by root exudates, decomposition of POC, or litter leachate laden with DOC. The focus on DOC contrasts with that on POC as the fraction of SOC most susceptible to management and soil structural disruption [16]–[22].

Given the previous research at HSB the objective of this study was to quantify SOC in various soil aggregate fractions under the different land uses to better understand the role of the aforementioned mechanisms in SOC stabilization [23], [24]. It was hypothesized that fractionation of SOC into POC and fine C (presumably adsorbed DOC) offers a means to address differences in the proposed mechanisms of carbon storage. In particular, it was hypothesized that if macro- and micro-aggregate protection of POC is the predominate mechanism increasing SOC then aggregate associated POC contents should be greater in land uses with higher SOC content. Conversely, if micro-scale fluxes of DOC are important to attaining maximum SOC storage capacity, then this should be recovered as fine C (<53 µm) in systems with significantly higher levels of SOC.

In addition to the POC and fine C fractionation, a soil moving experiment (SME) was established to determine the extent to which destructured soil can re-develop macroaggregate stability in the field under the contrasting land uses. This SME better represents land use attributes as opposed to laboratory incubations [5], [6]. It was hypothesized that differential recovery rates of macroaggregates in SME soil under the contrasting field conditions would reflect a greater abundance of organic binding agents as indexed by soil microbial biomass (i.e., bacteria and fungi). We hypothesized these rates would be greatest in forests due to an expectation of greater fungal abundance.

## Methods

### Site history

The Horseshoe Bend (HSB) agroecosystem experiment (33°57′N, 83°23′W) is a long-term comparison of conventional tillage (CT) and no-tillage (NT) treatments begun in 1978. The site is under the management of the Odum School of Ecology at The University of Georgia so no special permission was required for the current research and no endangered or protected species were involved in or negatively impacted by this research.

Based on a series of aerial photos, HSB had been cleared entirely of trees except for a narrow strip along the Oconee River at least since 1938 [7]. The site was used for pasture and forage production until 1965 when the Institute of Ecology acquired HSB for research [25]. The area underwent secondary succession in 1966 except for the future agroecosystem site, which was tilled to plant a crop of millet for a separate, earlier study [26]. Afterwards, the site went fallow and was used for a prescribed burn study and a N fertilization study [27], [28]. In 1978, the study area was divided into equal numbers of CT and NT 0.1 ha plots (n = 4 for each type), and the old-field vegetation was mowed [25].

### Site description

Horseshoe Bend is so named because it was established in a bend on the banks of the Oconee River and as such the site is very flat (<1% slope). The soils on this river terrace have previously been identified as clayey, kaolinitic, thermic, Rhodic Kanhapludults [29] but have recently been reclassified as a fine-loamy, kaolinitic, thermic, Typic Kanhapludalf based on new particle-size analysis data in the control section and measurements of base saturation (Table 1) that are >98% in all three treatments at 140 cm (i.e., 125 cm below the top of the kandic horizon). Average rainfall from 1945 to 2003 in Athens was 125 cm with a mean annual temperature of 16.4°C

**Table 1:** Soil chemical attributes (mean ±1SD) for the Horseshoe Bend Agroecosystem Experiment, Athens, GA.

Treatment	Depth	pHs^1^	C	N	P^2^	K	Ca	Mg	ECEC^3^	Bulk Density
	cm		--------g/kg--------		µg/g	----------------------------cmol_c_/kg----------------------------				g/cm^3^
Conventional	0–5	5.16±0.04	9.73±0.75	0.75±0.06	4.1±0.6	0.36±0.04	2.13±0.22	0.54±0.03	3.04±0.27	1.45±0.09
Till	5–15	4.90±0.04	6.99±0.58	0.58±0.05	2.2±0.4	0.25±0.03	1.93±0.13	0.49±0.02	2.72±0.18	1.61±0.03
	15–30	5.42±0.08	2.93±0.35	0.27±0.03	1.7±0.1	0.18±0.01	1.92±0.05	0.58±0.03	2.69±0.07	1.68±0.04
	30–50	5.69±0.06	1.84±0.12	0.20±0.02	0.7±0.2	0.12±0.01	1.89±0.07	0.58±0.01	2.58±0.06	1.64±0.03
	50–100	6.00±0.09	1.16±0.05	0.14±0.01	0.4±0.2	0.08±0.01	1.82±0.10	0.41±0.06	2.31±0.15	1.61±0.03
	100–150	6.05±0.06	0.85±0.04	0.10±0.00	0.4±0.1	0.04±0.00	1.87±0.05	0.38±0.07	2.29±0.11	
	150–200	5.98±0.09	0.54±0.02	0.06±0.00	0.4±0.0	0.03±0.01	1.20±0.07	0.33±0.09	1.56±0.12	
No Till	0–5	5.12±0.14	17.93±0.42	1.53±0.04	11.7±2.2	0.34±0.04	4.06±0.35	0.90±0.06	5.32±0.43	1.32±0.02
	5–15	5.09±0.12	6.92±0.31	0.54±0.03	4.3±1.2	0.27±0.05	2.31±0.13	0.57±0.03	3.18±0.17	1.50±0.06
	15–30	5.45±0.04	3.45±0.25	0.30±0.01	1.2±0.2	0.19±0.03	2.00±0.02	0.67±0.04	2.86±0.05	1.55±0.05
	30–50	5.74±0.06	2.14±0.13	0.21±0.01	0.5±0.1	0.14±0.02	1.97±0.10	0.69±0.04	2.80±0.10	1.60±0.05
	50–100	5.87±0.08	1.19±0.08	0.14±0.01	0.4±0.0	0.08±0.01	2.04±0.07	0.43±0.04	2.54±0.07	1.67±0.04
	100–150	5.93±0.05	0.98±0.07	0.11±0.00	0.3±0.0	0.04±0.01	1.99±0.10	0.30±0.07	2.32±0.06	
	150–200	5.93±0.05	0.64±0.06	0.07±0.01	0.3±0.0	0.03±0.00	1.48±0.15	0.27±0.11	1.77±0.19	
Forest	0–5	4.33±0.15	22.4±1.44	1.55±0.11	1.5±0.7	0.23±0.05	2.42±0.51	0.81±0.13	3.97±0.53	1.06±0.05
Succession	5–15	4.04±0.10	8.02±0.34	0.60±0.04	1.0±0.4	0.11±0.03	0.85±0.33	0.38±0.07	2.34±0.20	1.38±0.02
	15–30	4.38±0.06	3.52±0.18	0.30±0.01	0.5±0.1	0.06±0.01	1.34±0.19	0.49±0.02	2.44±0.14	1.65±0.02
	30–50	4.76±0.12	2.11±0.07	0.19±0.01	0.4±0.0	0.04±0.01	1.62±0.20	0.37±0.01	2.23±0.16	1.57±0.06
	50–100	5.19±0.14	1.40±0.21	0.14±0.01	0.4±0.1	0.03±0.00	1.46±0.09	0.37±0.04	1.89±0.08	1.63±0.04
	100–150	5.50 ±0.16	0.81 ±0.06	0.08±0.01	0.4±0.1	0.02±0.00	1.04±0.11	0.46±0.11	1.54±0.16	
	150–200	5.30 ±0.21	0.61 ±0.07	0.06±0.01	0.3±0.1	0.03±0.00	0.61±0.13	0.55±0.15	1.20±0.26	

N = 4 per treatment type. Soils were collected in 2009.

^1^ Salt pH in 0.01 M CaCl_2_.

^2^ Mehlich I extractable P and cations.

^3^ Effective cation exchange capacity by sum of cations method.

### Experimental design

When the site was originally sub-divided in 1978 the CT-NT treatments were assigned randomly in a completely randomized design [25]. Since the agroecosystem plots were in fallow from 1966, the current NT plots have not been tilled for forty-two years. Tillage has been performed twice annually in most years with moldboard plowing to a depth of 15 cm, followed by disking. In 1981, each agricultural plot was split into different winter crop treatments with winter rye (*Secale cereale*) grown on half of each plot and N-fixing crimson clover (*Trifolium incarnatum*) grown on the other half until 1984 and then again from 1989 to 2007 [25], [30]. From 1978–1998, summer crops have included sorghum (*Sorghum bicolor*), soybeans (*Glycine max*), corn (*Zea mays*), and kenaf (*Hibiscus cannabinus*). In 1999 to 2007, a second split-plot was assigned to the agroecosystem experiment for a comparison between summer crops of non-Bt and Bt-cotton (*Gossypium hirsutum* L.) [31]. Soil sampling in the current study sought to estimate an average effect of NT and CT on the measured properties by sampling equally among the winter cover split-plots before compositing; the effects of transgenic cotton on soil C and N were considered negligible, since earlier work showed no difference in decomposition rates between non-Bt and Bt-cotton residues [32].

In 2007, four secondary, hardwood forest plots bordering the tillage experiment were established. These areas represent a third treatment, referred to herein as forest succession (FS). Most likely, these afforested plots were not tilled in 1966, which means they essentially underwent a pasture to forest conversion. All FS plots are on the same terrace as the agroecosystem plots and were clear of forest from 1938 to 1967, as observed in the aerial photographs [7].

### Aggregate size fractions and stability

To quantify aggregate stability, four samples were obtained per plot at the end of the summer growing season in October 2007 using a 5 cm diameter corer attached to a slide-hammer to a depth of 28 cm. Each core was divided into 0–5, 5–15, and 15–28 cm and then composited by depth. Composite samples were passed through a 10 mm mesh by gently breaking the cores along planes of weakness before air-drying. These air-dried samples were dry-sieved by hand through 6.3, 4, 2, and 1 mm screens to obtain dry aggregate size classes. A separate subsample of the <1 mm dry aggregates was sieved on a 0.25 mm screen. The objective of dry-sieving was to determine the initial aggregate size distribution before wet-sieving [33]. Sub-samples of 50 g were then weighed out by re-combining all the dry aggregate size classes according to their proportional mass for the bulk soil. This ensured that each sub-sample accurately represented the bulk soil dry aggregate size distribution [34]. These 50 g sub-samples were then re-wetted via capillary action followed by wet-sieving with a modified Yoder apparatus [35], which permits complete recovery of all soil from each sample. The method obtains four wet aggregate size classes: (i) >2000 µm; (ii) 250–2000 µm; (iii) 53–250 µm; and (iv) <53 µm. Wet aggregate mean-weighted diameters (MWD) were calculated by multiplying the proportion of soil in each of these four aggregate size classes by the mid-point of the size class. The calculation of the dry MWD used the same size divisions as the wet MWD except no <53 µm fraction was included, since dry sieving material through a 53 µm sieve is not practical. The aggregate stability index was then calculated by dividing the wet MWD by the dry MWD; an index of 1 represents perfect structural stability.

### Soil organic C (SOC) fractionation and determination

Soil sub-samples were pulverized in a Spex 8200 ball-mill grinder (Metuchen, NJ) and total SOC and N were determined by dry combustion on a CE Elantech NC 1110 analyzer (Lakewood, NJ). Earlier mineralogical work on subsamples showed no evidence of carbonates so no pre-treatments were performed (Devine, unpublished data, 2008). To obtain particulate organic carbon (POC) and fine-C fractions, a 3–5 g subsample of each aggregate size fraction was dispersed by adding 15–25 mL of 0.5% Na-hexametaphosphate and shaking in 50 mL centrifuge tubes for 16 h on a reciprocal shaker at 200 rpm. The resulting soil slurry was washed through a 53 µm sieve using 0.5 L of DI water to fractionate the soil into: (1) sand+POC and (2) silt+clay+fine-C. A rubber policeman was used to aid in dispersion of stable soil aggregates on top of the 53 µm sieve when necessary. After drying both fractions in a forced-air oven at 60°C, fine-C and POC fractions were pulverized and C and N determined. A mass correction was made for the amount of Na-HMP recovered in the fine fraction prior to estimation of fine-C and N concentrations.

Since the <53 µm fraction does not contain sand, total SOC and POC are calculated on a sand-free basis for each aggregate size fraction in order to make comparisons among all the aggregate size classes [36]:
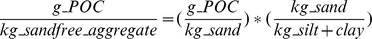


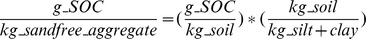



### Soil moving experiment

A soil moving experiment (SME) was carried out to determine the extent to which destructured soil re-develops aggregate stability among the different land uses. Soil moving or transplant experiments have been carried out to investigate soil processes including C accumulation or N cycling [37], [38]. In this study, destructured soil was utilized, as opposed to intact cores, which is similar to the use of sand or sieved soil used in root ingrowth core experiments [39]. First, CT soil from 0–15 cm was collected from all four plots. CT soil was utilized since previous research at HSB has measured this soil to have the least SOC and aggregate stability. Sole use of CT soil, however, also assumes that there is no inherent internal difference between destructured CT, NT, or FS soil and that differences during the experiment would result only from external differences due to land use. After collection soil was dried at 60°C in a forced-air oven, and crushed with a rolling pin to destroy larger macroaggregates and to pass a 1 mm sieve, referred to henceforth as “initial soil.” A 10 cm diameter auger was used to remove soil to 20 cm depth from four locations in each CT, NT, and FS plot at the end of July 2007. A PVC pipe of an equal diameter was placed as an insert in each hole to prevent the side walls from collapsing during filling. Each hole was then filled with the initial soil and uniformly tamped with a capped PVC pipe to a bulk density of approximately 1.4 g soil cm^−3^. Soil packing was tested with success in the laboratory by filling the same PVC pipe insert to the desired bulk density. The PVC insert was removed after packing, and the edge of the SME core marked with flags and aluminum nails. At the end of August 2008, the same 5 cm diameter corer previously used to sample aggregates was used to extract a smaller soil core within the 10 cm diameter SME core. All cores were located (some with the aid of a metal detector), divided into 0–5 and 5–15 cm depth classes, and composited by plot. In one FS plot, only two cores were sampled due to obvious mixing with native soil that had occurred from animal activity; in three other plots (two CT and one FS), only three cores were sampled due to obvious animal activity.

The same procedure as described above was used to measure aggregate stability, total SOC, and POC in the two depth classes. Microbial biomass C was also determined on field moist, replicate subsamples following the 24 hour chloroform fumigation—K_2_SO_4_ extraction procedure [40]. Extracts were analyzed for non-purgable organic carbon (NPOC) (Shimadzu TOC 5000, Columbia, Maryland, USA). Microbial biomass C (MBC) was estimated following Wu et al. [41]:
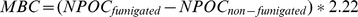



### Statistical analysis

All statistical analyses were completed with SAS 9.3.1 (SAS, Cary, NC). All data were checked for normality using Shapiro-Wilk and Kolmogorov-Smirnoff but no transformations were deemed necessary. Differences in soil properties were tested with a one-way ANOVA by each depth class and aggregate size combination with land use as the main effect (n = 4). Equality of variance among treatment classes was ensured with Levine's test. The same analysis was performed by each depth class and land use combination with aggregate size as the main effect. For the soil moving experiment where starting values of soil properties were known, changes in total SOC, POC, and fine-C were analyzed using a repeated measures ANOVA by depth class to investigate the effect of time and the land-use *x* time interaction. When the main effects were significant, post-hoc tests were performed using the LSMEANS statement in PROC GLM to evaluate the significance of mean separations with p-values adjusted by Tukey's honestly significant difference (α = 0.05).

## Results

### Aggregate size distributions and stability

**Figure 1:**
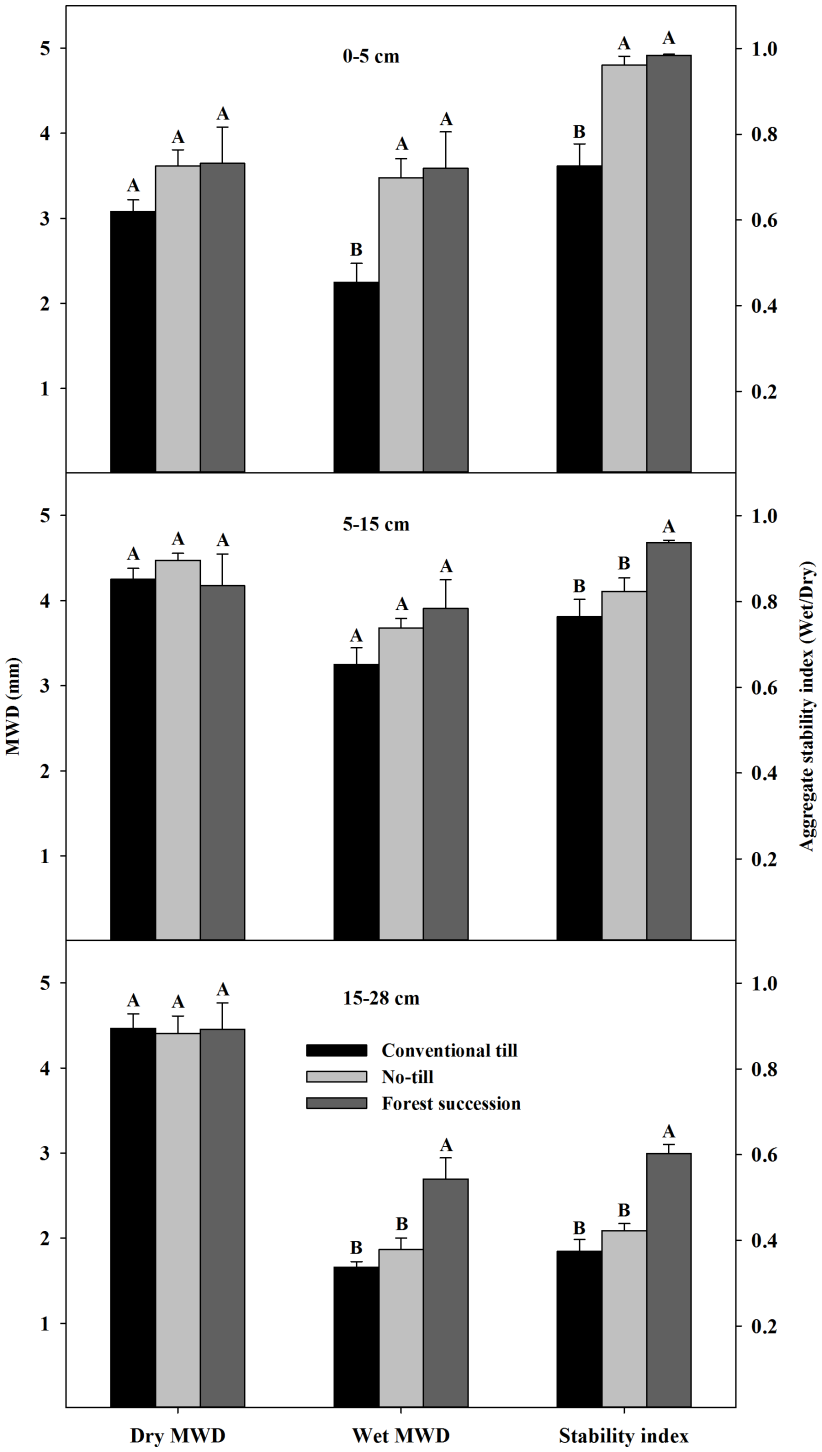
Dry-sieved and wet-sieved aggregates' mean weight diameter (MWD) (mean ± 1 SE) at the Horseshoe Bend Agroecosystem Experiment (n = 4), Athens, GA, USA, October 2007. The aggregate stability index is calculated by dividing the Wet MWD by the Dry MWD. Different letters indicate statistical significance between land uses within a depth class based on Tukey's HSD with a = 0.05. See also Table S1

**Figure 2:**
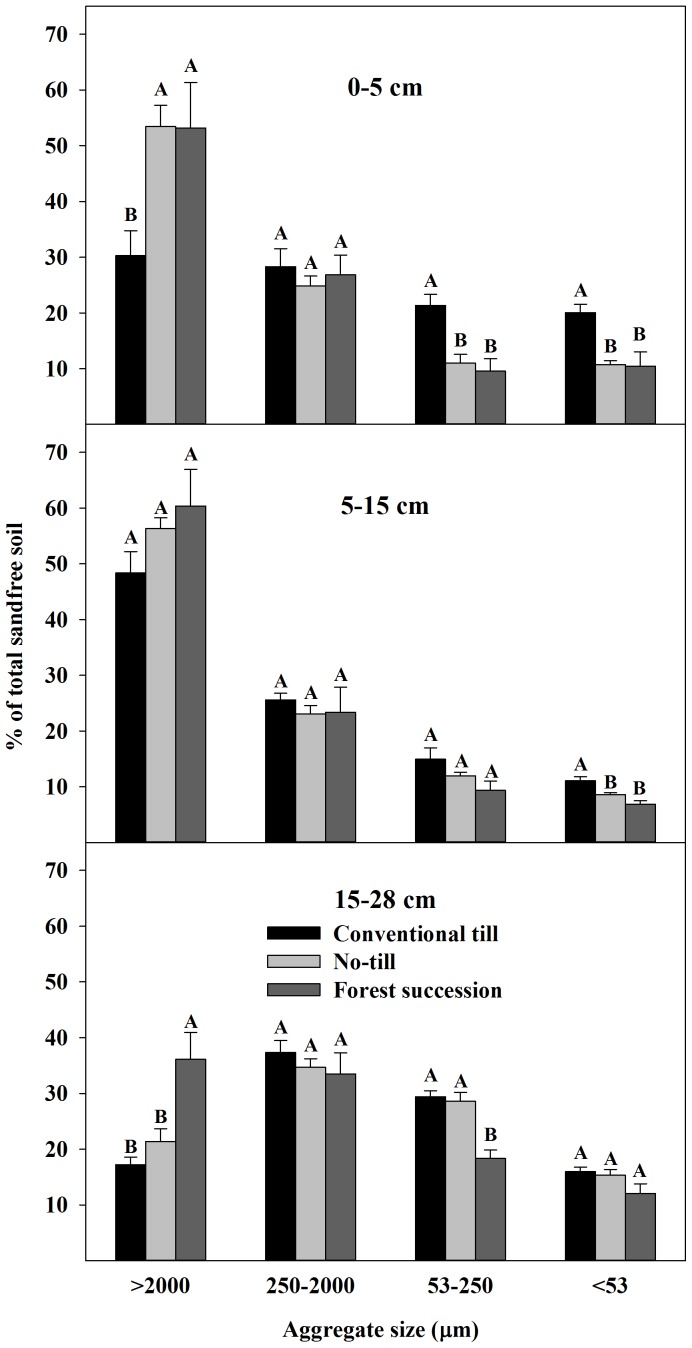
Distribution of water-stable aggregates on a sand-free soil basis (mean ±1 SE) at the Horseshoe Bend Agroecosystem Experiment (n = 4), Athens, GA, USA, October 2007. Different letters within an aggregate size class and depth class represent a significant difference between land uses (α = 0.05). See also Table S2.

Aggregate stability decreased significantly from FS and NT to CT in the upper 5 cm (Figure 1; see also Table S1). In the 5–28 cm layers FS soil was significantly more stable than both NT and CT (Figure 1). This response can also be seen as: (i) a higher percentage of wet-sieved, large macroaggregates (>2000 µm) in the FS and NT soils from 0–5 cm compared to CT, (ii) more large macroaggregates in FS from 15–28 cm compared to both CT and NT, and (iii) a higher proportion of fines (<53 µm) in 0–15 cm under CT compared to both NT and FS (Figure 2; see also Table S2).

### Land use effects on aggregate associated C

**Figure 3:**
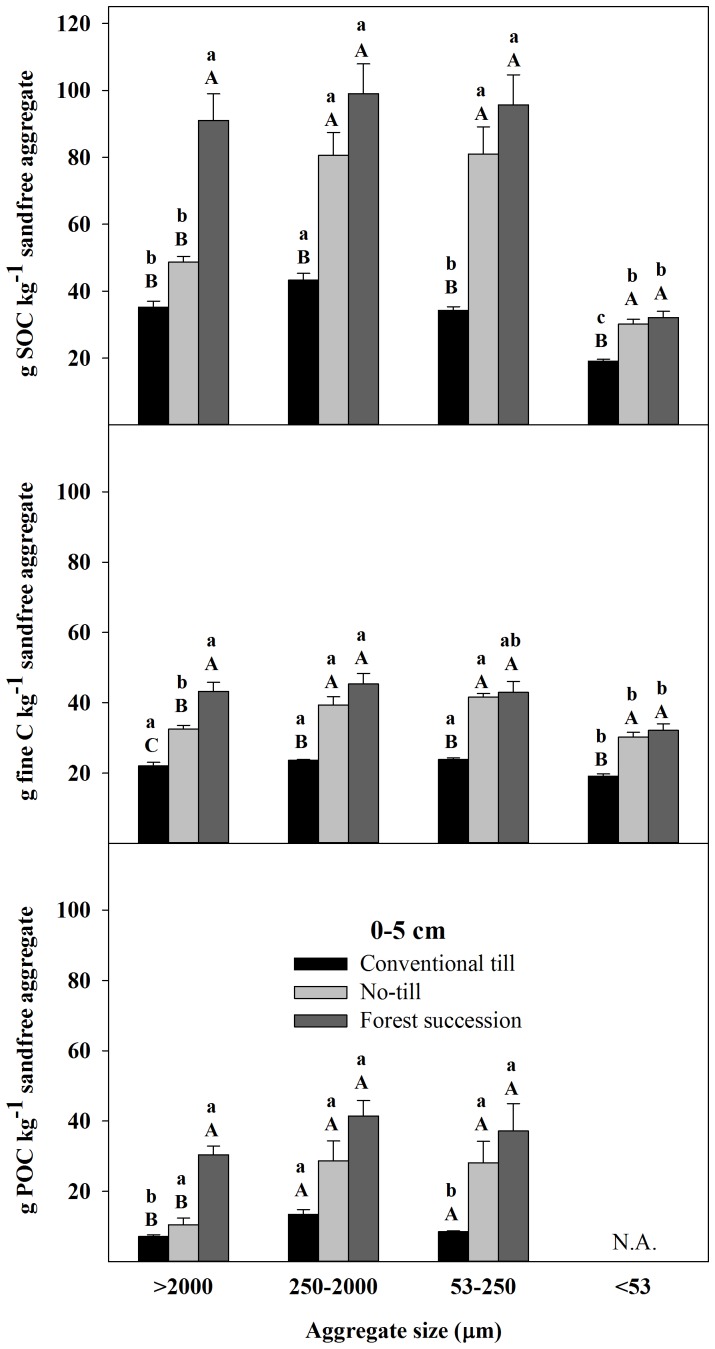
Total soil organic C (SOC), fine C (<53 µm), and particulate organic C (POC) (53–2000 µm) concentrations on a sand-free basis from 0–5 cm in four different aggregate size fractions (mean ±1 SE) in the Horseshoe Bend Agroecosystem Experiment (n = 4) Athens, GA, USA, October 2007. Different uppercase letters within an aggregate size class and C fraction represent a significant difference between land uses. Different lowercase letters within a C fraction and a land use represent a significant difference among aggregates size classes (α = 0.05). See also Table S3, part 1 and Table S3, part 2.

**Figure 4:**
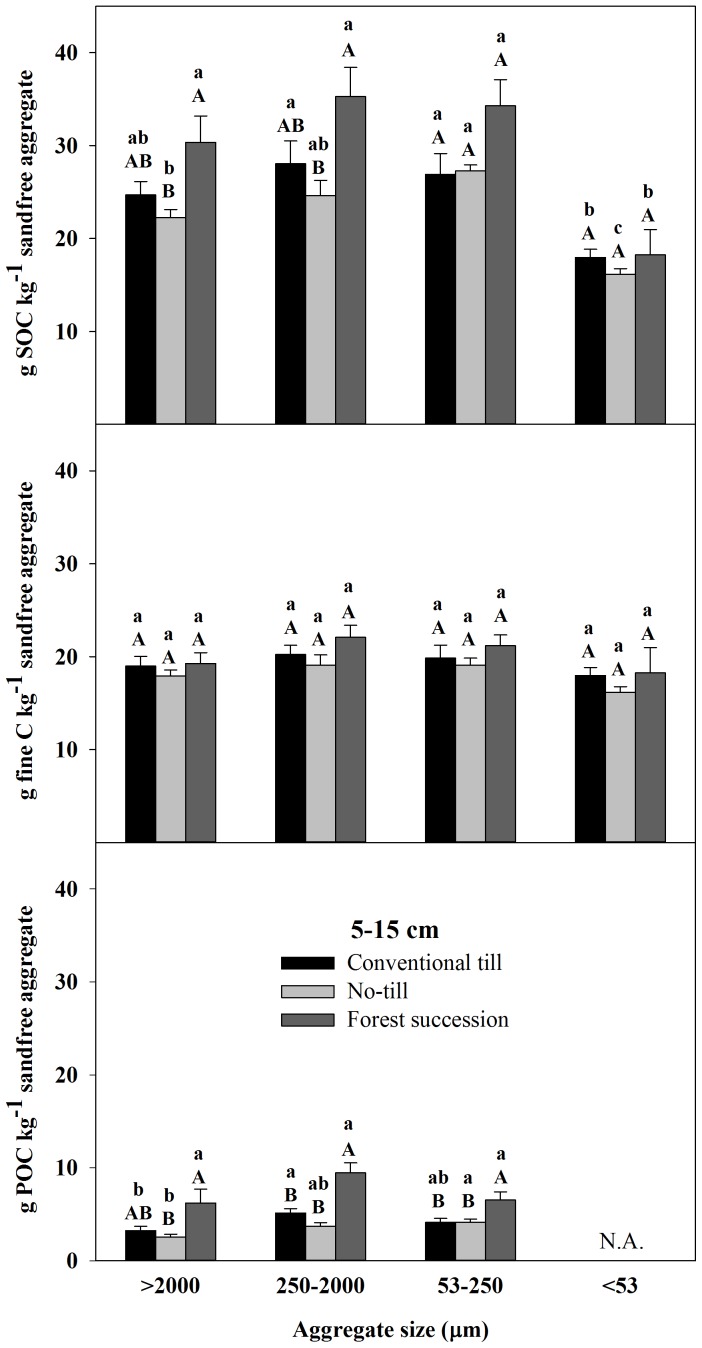
Total soil organic C (SOC), fine C (<53 µm), and particulate organic C (POC) (53–2000 µm) concentrations on a sand-free basis from 5–15 cm in four different aggregate size fractions (mean ±1 SE) in the Horseshoe Bend Agroecosystem Experiment (n = 4) Athens, GA, USA, October 2007. Different uppercase letters within an aggregate size class and C fraction represent a significant difference between land uses. Different lowercase letters within a C fraction and a land use represent a significant difference among aggregates size classes (α = 0.05). See also Table S4, part 1 and Table S4, part 2.

**Figure 5:**
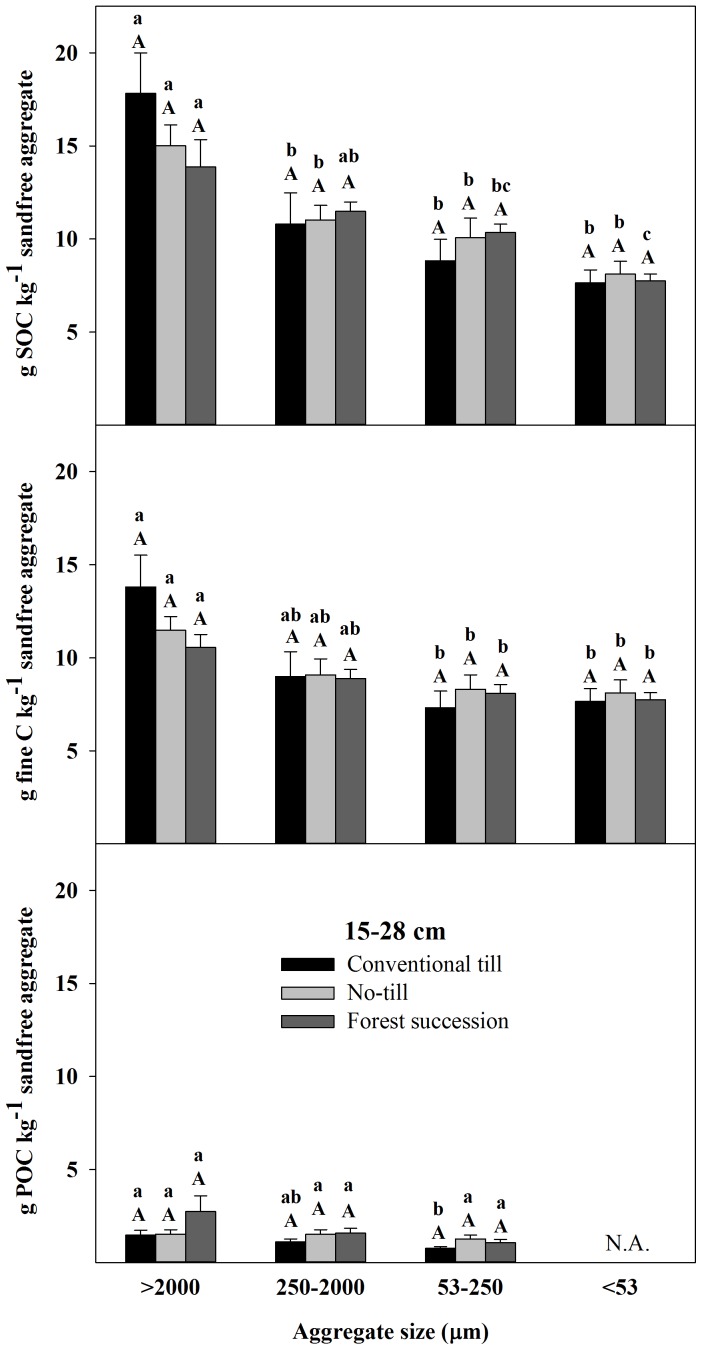
Total soil organic C (SOC), fine C (<53 µm), and particulate organic C (POC) (53–2000 µm) concentrations on a sand-free basis from 15–28 cm in four different aggregate size fractions (mean ±1 SE) in the Horseshoe Bend Agroecosystem Experiment (n = 4) Athens, GA, USA, October 2007. Different uppercase letters within an aggregate size class and C fraction represent a significant difference between land uses. Different lowercase letters within a C fraction and a land use represent a significant difference among aggregates size classes (α = 0.05). See also Table S5, part 1 and Table S5, part 2.

There were significant effects of land use on C fraction concentrations in all aggregate size classes from 0–5 cm and the majority of size classes from 5–15 cm. From 0–5 cm, NT and FS aggregate size fractions were significantly elevated for SOC and fine fractions compared to CT. In POC, increasing C from CT>NT>FS was evident in all aggregate sizes but significant at 0.05 for only the >2000 µm (Figure 3; see also Table S3, part 1). From 5–15 cm, there were no significant differences between CT and NT for any of the size class and C fraction combinations, while the two largest aggregate size classes were significantly elevated in FS with respect to NT for SOC and all three classes were greater for POC (Figure 4; see also Table S4, part 1). FS also exceeded CT but only in the small and micro aggregate fractions for POC. There were no significant differences for any of the size class and C fraction combinations from 15–28 cm (Figure 5; see also Table S5, part 1).

**Figure 6:**
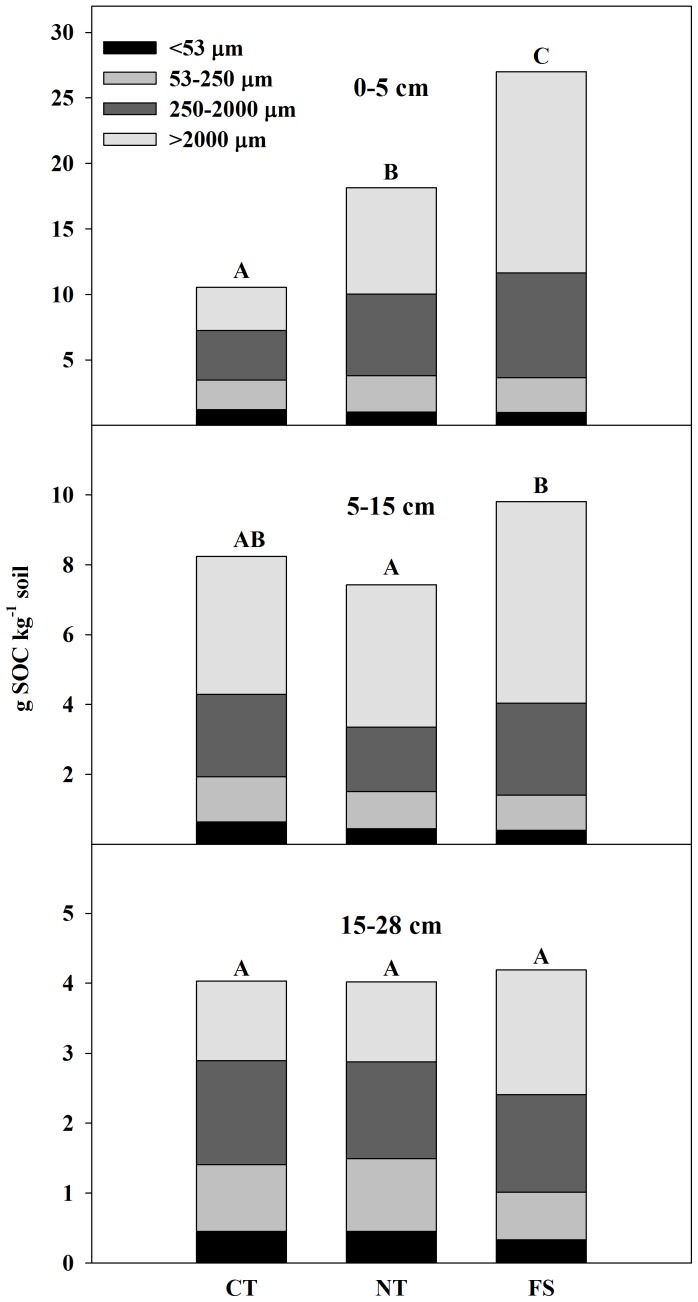
Contributions of four different aggregate size fractions to total soil organic C (SOC) concentrations. Different letters indicate a significant difference between land uses in a depth class (α = 0.05). Means of the sums of the four fractions are shown (n = 4) from Horseshoe Bend, Athens, GA, USA, October 2007. See also Table S6.

Overall, the majority of C at HSB under all land uses is contained in the water-stable macroaggregate fraction to a depth of at least 28 cm (Figure 6; see also Table S6). By summing the C contribution from each aggregate size fraction to calculate a C concentration for each depth class by treatment combination, differences in POC from 0–5 cm explained 34% of the total SOC difference between NT and CT, 43% of the total SOC difference between FS and CT, and 51% of the total SOC difference between FS and NT. From 5–15 cm, differences in POC explained 56% of the difference between FS and CT and 47% of the difference between FS and NT.

### Aggregate associated C within a land use

In terms of significant differences among the aggregate size classes within a land use and depth class, the most consistent result was that the <53 µm aggregate fraction had lower total SOC concentrations when expressed on a sand-free basis (Figures 3–5; see also Table S3, part 2, Table S4, part 2, and Table S5, part2). Under FS from 0–15 cm, there were no other significant differences among the size classes >53 µm (Figures 3 and 4) and there were no significant differences in POC among the aggregate size classes for any depth (Figures 3–5).

Under NT, in the upper 15 cm both the large macroaggregates and the <53 µm fractions were SOC depleted relative to the small macroaggregates (250–2000 µm) and microaggregates (Figures 3 and 4). This was similar under CT from 0–5 cm where the small macroaggregates (250–2000 µm) were significantly elevated in total SOC and POC compared to the other size fractions (Figure 3). Under CT from 5–15 cm, both the small macroaggregates and microaggregates were elevated in total SOC and POC compared to the large macroaggregates but only the small macroaggregates were significantly elevated compared to the large macroaggregates and only for POC (Figure 4). From 15–28 cm, the large macroaggregates were the most carbon rich under all land uses, having significantly greater total SOC and fine C concentrations compared to the <250 µm size fractions (Figure 5).

### Recovery of POC and fine C

Summation of POC and fine C compared to direct analysis of total SOC revealed that recoveries of POC and fine C were, on average, 86% from 0–5 cm, 88% from 5–15 cm, and 92% from 15–28 cm. A precipitate formed on the glass beakers used to dry the fine C slurry and was more difficult to remove in the beakers containing 0–5 cm samples, suggesting this may have been the source of the <100% recovery of C.

### Soil moving experiment (SME): Structural recovery, C change, and microbial biomass

The SME soil under FS had a significantly greater stability index compared to both CT and NT at 0–5 and 5–15 cm after one year (Table 2; see also Table S7). There were no significant differences in aggregate stability between CT and NT at either depth in the SME (Table 2). Overall, aggregate stability tended to be greater from 0–5 cm compared to 5–15 cm under NT and FS. This depth stratification did not occur under CT (Table 2).

**Table 2:** Aggregate stability (mean ±1 SD) at two depths at the end of a soil moving experiment (n = 4).

*Depth*	*Land use*	*Dry MWD* †	*Wet MWD*	*Stability index*
cm		-----------mm-----------		Wet/Dry
0–5	Conventional Till	4.3±0.4^a^	1.4±0.2^a^	0.33±0.06^a^
	No-Till	4.5±0.3^a^	1.9±0.3^a^	0.43±0.09^a^
	Forest Succession	3.9±0.3^a^	3.1±0.2^b^	0.79±0.03^b^
5–15	Conventional Till	4.8±0.6^a^	1.2±0.2^a^	0.26±0.08^a^
	No-Till	4.8±0.6^a^	1.0±0.2^a^	0.22±0.05^a^
	Forest Succession	3.6±0.5^b^	2.1±0.3^b^	0.60±0.10^b^

Conventional till soil was initially dried and crushed to pass a 1 mm sieve and then installed under three different land uses for a period of one year at Horseshoe Bend, Athens, GA, USA, July 2007–August 2008.

Different letters indicate statistical significance among treatment means within a depth class based on Tukey's HSD with α = 0.05. See also Table S7.

Mean-weighted diameter.

Repeated measures analysis showed a significant time *x* treatment interaction effect from 0–5 cm for total SOC (*p* = 0.03) and POC (*p* = 0.0004) but not fine C (*p* = 0.50), suggesting a significant departure from initial values of SOC and POC was dependent upon land use. The overall effect of time from 0–5 cm was significant for POC (*p* = 0.0007), which increased after one year. The effect of time was not significant for SOC (*p* = 0.27) or fine C (*p* = 0.35) for 0–5 cm, which means there was no clear directional change for these components compared to initial values across all land uses (Table 3: see also S8). A test of mean separation showed that total SOC from 0–5 cm was significantly elevated in the FS soil compared to CT (*p* = 0.08) and compared to NT (*p* = 0.03) (Table 3). POC was also significantly elevated from 0–5 cm in the FS soil compared to both CT and NT (*p*<0.002) (Table 3; see also Table S8). From 5–15 cm, declines in C were observed after one year. The effect of time was significant for SOC (*p*<0.0001), POC (*p* = 0.0004), and fine C (*p*<0.0001), meaning a significant decrease in these components from the initial values had occurred (Table 3). The time *x* treatment interaction was significant for POC at *p* = 0.099 possibly reflecting a lower net loss of POC from 5–15 cm under FS (Table 3). The interaction was not significant for SOC (*p* = 0.85) or fine C (*p* = 0.89), since there was a more uniform decrease in these components from the initial value under all the land uses.

**Table 3:** Total soil organic C (SOC), particulate organic C (POC) and microbial biomass C (MBC) (mean ±1 SD) at two depths at the end of a soil moving experiment (n = 4).

*Land use*	*Depth*	*SOC*	*POC*	*Fine C*	*MBC*
	-cm-	---------------g kg^−1^ soil--------------			--mg kg^−1^--
Initial	0–5	9.2±0.2	1.8±0.1	7.5±0.1	NA
Conventional till		9.1±0.3^a^	2.0±0.2^a^	7.2±0.2^a^	397±27^a^
No-till		8.7±0.2^a^	1.7±0.1^a^	7.0±0.2^a^	366±30^a^
Forest succession		10.9±1.7^b^	3.3±0.6^b^	7.6±1.2^a^	422±72^a^
Initial	5–15	9.2±0.2	1.8±0.1	7.5±0.1	NA
Conventional till		8.3±0.2^a^	1.5±0.1^a^	6.8±0.2^a^	317±25^a^
No-till		8.3±0.2^a^	1.4±0.1^a^	6.8±0.3^a^	274±13^b^
Forest succession		8.4±0.6^a^	1.7±0.2^a^	6.7±0.5^a^	305±16^ab^

Conventional till soil was initially dried and crushed to pass a 1 mm sieve and then installed under the three different land uses for a period of one year at Horseshoe Bend, Athens, GA, USA, July 2007–August 2008.

Different letters indicate statistical significance between treatment means within a depth class based on Tukey's HSD with α = 0.05. See also Table S8.

SME microbial biomass carbon (MBC), like aggregate stability and total SOC from 0–5 cm, was elevated in FS relative to the agroecosystem soils, though not significantly (*p*>0.27; Table 3). Overall, there were no significant differences among the land uses from 0–5 cm in terms of MBC concentrations. CT MBC were significantly elevated compared to NT from 5–15 cm (*p* = 0.02). For all three land uses, MBC tended to be higher from 0–5 cm compared to 5–15 cm (Table 3).

## Discussion

### Changes in soil structure and SOC after 30 years of different management

Earlier work from HSB reported that the three decades of divergent land use have led to 20% greater SOC content in the FS (61.5±4.3 Mg ha^−1^) and NT (60.0±4.5 Mg ha^−1^) soil profiles (0–2 m) relative to CT (51.9±4.9 Mg ha^−1^) [7]. SOC content differences in the 0–5 cm layer accounted for 56% of the 0–2 m mean content difference between FS and CT and 66% of the 0–2 m content difference between NT and CT. These increases in SOC at the surface under FS and NT are linked to enhanced aggregate stability.

The increase in structural stability under NT compared to CT was limited to a depth of 5 cm, while the FS soil demonstrated significantly greater stability compared to CT to a depth of 28 cm and compared to NT from 5–28 cm (Figure 1). Previous work at HSB on aggregate size fractions showed that all aggregates >106 µm from 0–5 cm were significantly more stable in NT compared to CT and from 5–15 cm the large macroaggregates (>2000 µm) were more stable in NT [23]. In the current study, the difference in aggregate stability between NT and FS from 5–15 cm is evident in the aggregate stability metric (Figure 1). The stability metric had lower coefficients of variation within a land-use than did either the dry or wet mean-weighted diameter, indicating this metric may be more sensitive to the effects of land use on soil structure than analysis of aggregate size distributions produced from one methodology (i.e., wet or dry sieving).

Significant differences in SOC were evident among the land uses in different aggregate size fractions. Both NT and FS generally had elevated concentrations in all fractions compared to CT from 0–5 cm with increases in POC and fine C each explaining 40–50% of concentration differences (Figure 3). With respect to our initial hypothesis this relatively equal contribution indicates that both stabilization of POC within microaggregates formed in stable macroaggregates [13], [42], and flux of DOC into unsaturated micropores [15] might play a role in soil carbon accumulation. The effect of tillage could disrupt either of these processes and afforestation did not favor either process in the 0–5 cm surface soil.

The FS soil also had elevated concentrations of SOC from 5–15 cm compared to NT for macro- and microaggregates and compared to CT for microaggregates with the increases in POC explaining 35–60% of the concentration differences (Figure 4). For CT and NT there were no differences in SOC concentrations among the aggregate size fractions from 5–15 cm, which has been found previously at HSB [6], [23], [24], [29]. Below 15 cm differences in aggregate stability were evident but significant differences in SOC fractions among the aggregate size fractions were not evident (Figure 5). This demonstrates that enhanced aggregate stability is not always linked to increased stabilization and storage of SOC.

Within the FS soil from 0–15 cm, only the wet-sieved fine fraction (<53 µm) had a significantly lower C concentration than the other fractions within each depth class (Figures 3 and 4), highlighting the importance of POC to increasing total SOC under afforestation. In both agroecosystems, large macroaggregates have significantly lower proportions of POC compared to small macroaggregates at all depths (Table 4; see also Table S9) and most of the difference in SOC concentration at 0–5 cm and 5–15 cm is accounted for by differences in macroaggregate C (Figure 6). As such, most of the C difference in the agroecosystem compared to the FS soil occurs at the macroaggregate level.

**Table 4:** The proportion of particulate organic carbon (POC) within aggregate fractions and whole soil (mean ±1 SD).

*Land use*	*Depth*	*Large macroaggregates*	*Small macroaggregates*	*Microaggregates*	*Whole soil* 1
	--cm--	------------------------------------% POC------------------------------------			
CT	0–5	20.2±1.3^a^	31.0±4.2^a^	24.7±0.6^a^	22.6±0.9^a^
NT		21.3±6.3^a^	34.8±7.9^a^	33.7±7.3^a^	26.9±6.6^a^
FS		33.8±5.7^b^	41.8±4.6^a^	38.1±9.8^b^	35.5±5.1^b^
CT	5–15	13.0±2.4^ab^	18.4±1.9^a^	15.3±1.2^ab^	13.8±1.8^a^
NT		11.4±2.6^b^	14.9±2.6^a^	15.0±2.2^a^	12.1±2.3^a^
FS		19.7±6.4^a^	26.9±4.7^b^	18.8±2.0^b^	20.4±5.1^b^
CT	15–28	8.2±0.9^a^	10.4±1.2^a^	8.8±0.8^a^	8.2±0.5^a^
NT		10.1±2.0^a^	13.6±2.8^a^	12.5±1.9^a^	10.8±1.2^a^
FS		20.0±13.6^a^	13.7±3.5^a^	10.3±2.7^a^	15.1±6.7^a^

Horseshoe Bend, Athens, GA, USA, October 2007.

Different letters indicate statistical significance between treatment means within a depth class and aggregate class based on Tukey's HSD with α = 0.05. See also Table S9.

^1^ Calculated by summation of individual fractions.

In previous research at HSB, Beare et al. [5] observed the highest C concentrations in the large NT microaggregates (106–250 µm) compared to the macroaggregates from 0–5 cm and speculated that this was due to formation of microaggregates around decomposing residues in stable macroaggregates, followed by their release upon macroaggregate breakdown, as originally formulated by Oades [12]. Work that followed this at HSB also found the highest C concentrations in a broader 53–250 µm microaggregate class from 0–5 cm in NT [6], but this result was not found more recently [24] or in the present work, in which small macroaggregates were found to have similar C concentrations compared to microaggregates (Figure 3). These contrasting results at the same study site may be due to methodological differences in aggregate sieving. Beare et al. [5] used tension-wetted field-moist soil before wet-sieving and Bossuyt et al. [6] used capillary-wetted field moist soil before wet-sieving, whereas Arce Flores and Coleman [24] and the present work air-dried soils for storage and then capillary-wetted soils before wet-sieving. Beare and Bruce [43] clearly demonstrated that aggregate size distributions depend upon both pre-treatment and sieving methodologies.

Finally, aggregate hierarchy theory, which suggests that inter-microaggregate organic matter should increase C concentration of stable macroaggregates compared to microaggregates, is only evident in the 15–28 cm depth class (Figures 3–5). In all the 0–15 cm soils, large macroaggregates (>2000 µm) were relatively depleted in total SOC compared to the small macroaggregates (250–2000 µm) and microaggregates (53–250 µm). The method for isolating water-stable aggregates used repeatedly at HSB may not be forceful enough to isolate large macro-aggregates of higher C concentration in the upper 15 cm. If a more forceful fractionation procedure was used, such as sonication or slaking, then perhaps low C macroaggregates would be dispersed and their particles transferred to smaller fractions. Previous research has also shown that wet-sieving of pre-wetted soil does not support aggregate hierarchy theory, as was done in this study, while slaking dry soil does [11]. Also, aggregate hierarchy has been shown to be less apparent in soils dominated by 1∶1 clays with high oxide contents like at HSB compared to soils dominated by 2∶1 clays with low oxide contents [44], [45].

### Soil moving experiment

Significant differences in aggregate stability were evident after moving crushed and sieved (1 mm) soil from the CT plots back to each land use for both the 0–5 and 5–15 cm depth classes after one year (Table 2), suggesting that water-stable macroaggregates can be quickly formed under the different land uses but most quickly under FS, a result consistent with our initial hypothesis.

Similar rapid changes in soil structure were observed in a soil moving experiment in the tropics where larger blocks of soil were transferred from forest to pasture and vice-versa to study the effects of an invasive earthworm species [46]. The effects of different drying-wetting cycles, root densities, and levels of microbial activity are also known to affect soil structural development over time periods of days to a few years [47], [48], so the rapid, differential development of aggregate stability under different land uses is consistent with previous and current observations.

Since forest soils are fungal-dominated systems, any observed difference in aggregate stability was hypothesized to result from more rapid binding by fungal hyphae under FS but no significant differences were observed in MBC between land uses (Table 3). The chloroform-incubation method for MBC is related to direct counts of fungal and bacterial cells [49] but the method has failed to efficiently extract fungal biomass from some forest soils [50]. On the other hand, previous work at HSB has shown a sensitivity of the chloroform-incubation method to treatments since both MBC and fungal hyphae lengths significantly decreased after application of fungicide to a mixed meadow that had been recently plowed at HSB [51]. Furthermore, the importance of fungal hyphae in stabilizing aggregates was demonstrated in the agroecosystems by treating sub-plots with fungicides and observing declines in aggregate size distributions [29]. As such, further efforts will be needed to conclusively demonstrate the role of fungal hyphae in aggregate formation in FS at HSB.

Along with aggregate stability, total SOC also increased significantly from 0–5 cm under FS from 9.2 g SOC kg^−1^ soil to 10.8 g SOC kg^−1^ soil after one year. This increase in SOC could, however, be explained by mixing of a relatively small portion of the *in situ* FS soil SOC (9–22%) with the SME soil. In contrast, however, the significant increase in POC from 1.8 g POC kg^−1^ soil to 3.3 g POC kg^−1^ soil, which explains 90% of the increase in SOC, cannot be explained by *in situ* soil mixing since POC in 0–5 cm *in situ* forest soil accounts for only 35% of total SOC. The observed increase, which is equal to an increased storage of approximately 1 Mg C ha^−1^, could result partly from leaf litter inputs under FS that are estimated to be 2 Mg C ha^−1^ yr^−1^ (Markewtiz, 2009, unpublished data) or annual fine root production that has been reported to range globally from 0.3–8.2 Mg C ha^−1^ yr^−1^ [52]. Under a simulated no-till experiment with a Montana Hapludoll 14C labeled SOC in aggregates was derived predominantly from fine root litter [42], [53].

Finally, decreases in SOC compared to the initial concentration from 5–15 cm under all three land uses showed that C mineralization exceeded new C inputs in this sub-surface layer. This enhanced decomposition was possibly triggered by the loss of macroaggregate structure from crushing the soil before its installation. Interestingly, decreases in POC concentrations explained less than half (11–36%) of the loss in total SOC under all three land uses, suggesting that fine C was more susceptible to decomposition after structural disruption. This reduction occurred even though the soil used in the moving experiment was CT soil in which macroaggregate protection of SOC had already been impacted during 30 years of continuous moldboard plowing.

## Conclusions

Changes in aggregate size distributions after 30 years of differing land management were only clearly evident to a depth of 5 cm between conventional tillage (CT) and no-tillage (NT) systems. When adjacent land undergoing forest succession (FS) was added to the comparison, structural changes were evident to 28 cm. Changes in SOC concentration and composition occurred along with changes in structural stability to a depth of 15 cm, consistent with a reduced capacity for tilled soil to physically protect organic matter from decomposition. Although differences in stability were evident from 15–28 cm, no significant difference in SOC concentration was observed among the land uses, indicating that increased aggregate stability is not always linked to an increase in SOC storage. SOC results lend support to two different models of how SOC becomes physically protected in systems lacking mechanical disturbance, one that highlights the importance of particulate organic carbon (POC) and the other dissolved organic carbon (DOC). Since POC contributed only about half of the differences in SOC between land uses, it is possible that micro-scale fluxes of DOC are also playing an important role in maintaining higher fine-C concentrations in soils with greater pore connectivity, such as the NT and FS soils in this study. The importance of macroaggregate stability for the protection of both POC and fine C was also demonstrated by the destruction of large macroaggregates in a soil moving experiment in which losses of C from 5–15 cm were observed under all land uses after 1 yr. The SME experiment also demonstrated that the recovery of water-stable macroaggregates can be rapid, since significant increases in stability under FS were observed in the experiment to 15 cm. Finally, a gain of 1 Mg C ha^−1^ was detected in the 0–5 cm soil layer under FS during the SME, 90% of which could be accounted for by a gain in POC. The stabilization of this POC may have been enhanced by recovery of water-stable aggregates under FS.

## Supporting Information

Table S1 ANOVA results for Figure 1. ANOVA table reports tests of significance among Land Uses (conventional tillage, no tillage, forest succession) by aggregate attribute (Dry mean weighted diameter, Wet mean weighted diameter, Aggregate Stability {wet/dry}) and soil depth (0–5, 5–15, 15–28 cm).
(DOCX)

Table S2 ANOVA results for Figure 2. ANOVA table reports tests of significance among Land Uses (conventional tillage, no tillage, forest succession) by aggregate size fraction (>2000, 250–2000, 53–250, and <53 µm) and soil depth (0–5, 5–15, 15–28 cm).
(DOCX)

Table S3 1. ANOVA results for Figure 3 in soil depth 0–5 cm for land use. ANOVA table reports tests of significance among land uses (CT, NT, and FS) within a carbon fraction (Soil organic carbon, particulate organic carbon, fine carbon) and size class (>2000, 250–2000, 53–250, and <53 µm). 2. ANOVA results for Figure 3 in soil depth 0–5 cm for aggregate size class. ANOVA table reports tests of significance among size classes (2000, 250–2000, 53–250 and <53 µm) within a carbon fraction and land use. Note for POC the test is only for three size classes (2000, 250–2000, and 53–250 µm).
(DOCX)

Table S4 1. ANOVA results for Figure 4 in soil depth 5–15 cm for land use. ANOVA table reports tests of significance among land uses (CT, NT, and FS) within a carbon fraction and size class. 2. ANOVA results for Figure 4 in soil depth 5–15 cm for aggregate size class. ANOVA table reports tests of significance among size classes (2000, 250–2000, 53–250 and <53 µm) within a carbon fraction and land use.
(DOCX)

Table S5 1. ANOVA results for Figure 5 in soil depth 15–28 cm for land use. ANOVA table reports tests of significance among land uses (CT, NT, and FS) within a carbon fraction and size class. 2. ANOVA results for Figure 5 in soil depth 15–28 cm for aggregate size class. ANOVA table reports tests of significance among size classes (2000, 250–2000, 53–250 and <53 µm) within a carbon fraction and land use.
(DOCX)

Table S6 ANOVA results for Figure 6. ANOVA table reports tests of significance among Land Uses (conventional tillage, no tillage, forest succession) by soil depth (0–5, 5–15, 15–28 cm) for the sum of C contents in all aggregate fractions.
(DOCX)

Table S7 ANOVA results for Table 2. ANOVA table reports tests of significance among Land Uses (conventional tillage, no tillage, forest succession) by aggregate attribute (Dry mean weighted diameter, Wet mean weighted diameter, Aggregate Stability {wet/dry}) and soil depth (0–5, 5–15 cm).
(DOCX)

Table S8 ANOVA results for Table 3. ANOVA table reports tests of significance among Land Uses (conventional tillage, no tillage, forest succession) by soil carbon component (soil organic carbon, particulate organic carbon, fine carbon, microbial biomass carbon) and soil depth (0–5, 5–15 cm).
(DOCX)

Table S9 ANOVA results for Table 4. ANOVA table reports tests of significance among Land Uses (conventional tillage, no tillage, forest succession) by aggregate size fraction (>2000, 250–2000, 53–250, and <53 µm) and soil depth (0–5, 5–15, 15–28 cm).
(DOCX)
