# Supplementary material for: Soil Aggregates and Associated Organic Matter under Conventional Tillage, No-Tillage, and Forest Succession after Three Decades
Source: PLoS One. 2014 Jan 20;9(1):e84988. doi: 10.1371/journal.pone.0084988 (PMC3896348; doi:10.1371/journal.pone.0084988)
Supplement: Table S2 — ANOVA results for Figure 2. ANOVA table reports tests of significance among Land Uses (conventional tillage, no tillage, forest succession) by aggregate size fraction (>2000, 250–2000, 53–250, and <53 µm) and soil depth (0–5, 5–15, 15–28 cm). (DOCX) [file pone.0084988.s002.docx]

Table S2: ANOVA results for Figure 2. ANOVA table reports tests of significance among Land Uses (conventional tillage, no tillage, forest succession) by aggregate size fraction (>2000, 250-2000, 53-250, and <53 µm) and soil depth (0-5, 5-15, 15-28 cm).

| *Agg Size* | *Depth* | *Source* | *DF* | *SS* | *M1* | *F* | *Pr>F* |
| --- | --- | --- | --- | --- | --- | --- | --- |
| >2000 | 0-5 | Model | 2 | 1413 | 707 | 5.29 | 0.030 |
|  |  | Error | 9 | 1202 | 134 |  |  |
|  |  | Corrected Total | 11 | 2615 |  |  |  |
|  | 5-15 | Model | 2 | 297.0 | 148.5 | 1.80 | 0.221 |
|  |  | Error | 9 | 743.7 | 82.6 |  |  |
|  |  | Corrected Total | 11 | 1040.7 |  |  |  |
|  | 15-28 | Model | 2 | 787.1 | 392.5 | 9.75 | 0.006 |
|  |  | Error | 9 | 362.3 | 40.3 |  |  |
|  |  | Corrected Total | 11 | 1147.3 |  |  |  |
|  |  |  |  |  |  |  |  |
| 250-2000 | 0-5 | Model | 2 | 24.0 | 12.0 | 0.34 | 0.720 |
|  |  | Error | 9 | 315.9 | 35.1 |  |  |
|  |  | Corrected Total | 11 | 339.9 |  |  |  |
|  | 5-15 | Model | 2 | 15.2 | 7.58 | 0.24 | 0.790 |
|  |  | Error | 9 | 281.7 | 31.3 |  |  |
|  |  | Corrected Total | 11 | 296.9 |  |  |  |
|  | 15-28 | Model | 2 | 31.4 | 15.7 | 0.56 | 0.592 |
|  |  | Error | 9 | 254.0 | 28.2 |  |  |
|  |  | Corrected Total | 11 | 285.4 |  |  |  |
|  |  |  |  |  |  |  |  |
| 53-250 | 0-5 | Model | 2 | 331.8 | 165.9 | 11.03 | 0.004 |
|  |  | Error | 9 | 135.4 | 15.0 |  |  |
|  |  | Corrected Total | 11 | 467.2 |  |  |  |
|  | 5-15 | Model | 2 | 62.2 | 31.1 | 3.14 | 0.092 |
|  |  | Error | 9 | 89.1 | 9.9 |  |  |
|  |  | Corrected Total | 11 | 151.3 |  |  |  |
|  | 15-28 | Model | 2 | 304.8 | 152.4 | 18.98 | 0.001 |
|  |  | Error | 9 | 72.3 | 8.0 |  |  |
|  |  | Corrected Total | 11 | 377.1 |  |  |  |
|  |  |  |  |  |  |  |  |
| <53 | 0-5 | Model | 2 | 239.8 | 119.9 | 9.78 | 0.006 |
|  |  | Error | 9 | 110.3 | 12.3 |  |  |
|  |  | Corrected Total | 11 | 350.2 |  |  |  |
|  | 5-15 | Model | 2 | 35.3 | 17.6 | 13.74 | 0.002 |
|  |  | Error | 9 | 11.6 | 1.28 |  |  |
|  |  | Corrected Total | 11 | 46.8 |  |  |  |
|  | 15-28 | Model | 2 | 35.2 | 17.6 | 2.77 | 0.116 |
|  |  | Error | 9 | 57.2 | 6.4 |  |  |
|  |  | Corrected Total | 11 | 92.3 |  |  |  |
