# Supplementary material for: Soil Aggregates and Associated Organic Matter under Conventional Tillage, No-Tillage, and Forest Succession after Three Decades
Source: PLoS One. 2014 Jan 20;9(1):e84988. doi: 10.1371/journal.pone.0084988 (PMC3896348; doi:10.1371/journal.pone.0084988)
Supplement: Table S3 — 1. ANOVA results for Figure 3 in soil depth 0–5 cm for land use. ANOVA table reports tests of significance among land uses (CT, NT, and FS) within a carbon fraction (Soil organic carbon, particulate organic carbon, fine carbon) and size class (>2000, 250–2000, 53–250, and <53 µm). 2. ANOVA results for Figure 3 in soil depth 0–5 cm for aggregate size class. ANOVA table reports tests of significance among size classes (2000, 250–2000, 53–250 and <53 µm) within a carbon fraction and land use. Note for POC the test is only for three size classes (2000, 250–2000, and 53–250 µm). (DOCX) [file pone.0084988.s003.docx]

Table S3-1. ANOVA results for Figure 3 in soil depth 0-5 cm for land use. ANOVA table reports tests of significance among land uses (CT, NT, and FS) within a carbon fraction (soil organic carbon, particulate organic carbon, fine carbon) and size class (>2000, 250-2000, 53-250, and <53 µm).

| *C fraction* | *Size* | *Source* | *DF* | *SS* | *M1* | *F* | *Pr>F* |
| --- | --- | --- | --- | --- | --- | --- | --- |
| SOC | >2000 | Model | 2 | 6768 | 3384 | 36.5 | <0.0001 |
|  |  | Error | 9 | 835 | 92.8 |  |  |
|  |  | Corrected Total | 11 | 7604 |  |  |  |
|  | 250-2000 | Model | 2 | 6450 | 3225 | 18.6 | 0.0006 |
|  |  | Error | 9 | 1562 | 173 |  |  |
|  |  | Corrected Total | 11 | 8012 |  |  |  |
|  | 53-250 | Model | 2 | 8212 | 4106 | 20.9 | 0.0004 |
|  |  | Error | 9 | 1768 | 196.5 |  |  |
|  |  | Corrected Total | 11 | 9981 |  |  |  |
|  | <53 | Model | 2 | 394.7 | 197.3 | 24.8 | 0.0002 |
|  |  | Error | 9 | 71.7 | 7.97 |  |  |
|  |  | Corrected Total | 11 | 466.4 |  |  |  |
|  |  |  |  |  |  |  |  |
| Fine C | >2000 | Model | 2 | 896 | 448 | 37.2 | <0.0001 |
|  |  | Error | 9 | 108.5 | 12.1 |  |  |
|  |  | Corrected Total | 11 | 1005 |  |  |  |
|  | 250-2000 | Model | 2 | 1011 | 505.9 | 26.4 | 0.0002 |
|  |  | Error | 9 | 172.2 | 19.1 |  |  |
|  |  | Corrected Total | 11 | 1184 |  |  |  |
|  | 53-250 | Model | 2 | 908.4 | 454.2 | 31.2 | <0.0001 |
|  |  | Error | 9 | 131.0 | 14.6 |  |  |
|  |  | Corrected Total | 11 | 1039 |  |  |  |
|  | <53 | Model | 2 | 394.7 | 197.3 | 24.8 | 0.0002 |
|  |  | Error | 9 | 71.7 | 7.97 |  |  |
|  |  | Corrected Total | 11 | 466.4 |  |  |  |
|  |  |  |  |  |  |  |  |
| POC | >2000 | Model | 2 | 1265 | 632 | 47.4 | <0.0001 |
|  |  | Error | 9 | 120.0 | 0.318 |  |  |
|  |  | Corrected Total | 11 | 1385 |  |  |  |
|  | 250-2000 | Model | 2 | 1567 | 783 | 11.2 | 0.0036 |
|  |  | Error | 9 | 630 | 70.0 |  |  |
|  |  | Corrected Total | 11 | 2197 |  |  |  |
|  | 53-250 | Model | 2 | 1720 | 860.2 | 6.6 | 0.017 |
|  |  | Error | 9 | 1173 | 130.3 |  |  |
|  |  | Corrected Total | 11 | 2893 |  |  |  |
|  | <53 | Model |  |  |  |  |  |
|  |  | Error |  |  |  |  |  |
|  |  | Corrected Total |  |  |  |  |  |

Table S3-2. ANOVA results for Figure 3 in soil depth 0-5 cm for aggregate size classes. ANOVA table reports tests of significance among size classes (2000, 250-2000, 53-250 and <53µm) within a carbon fraction and land use. Note for POC the test is only for three size classes (2000, 250-2000, and 53-250 µm)

| *C fraction* | *Land Use* | *Source* | *DF* | *SS* | *M1* | *F* | *Pr>F* |
| --- | --- | --- | --- | --- | --- | --- | --- |
| SOC | CT | Model | 3 | 1222 | 407.5 | 46.6 | <0.0001 |
|  |  | Error | 12 | 105.0 | 8.75 |  |  |
|  |  | Corrected Total | 15 | 1327 |  |  |  |
|  | NT | Model | 3 | 7514.8 | 2505.0 | 21.15 | <0.0001 |
|  |  | Error | 12 | 1421.1 | 118.4 |  |  |
|  |  | Corrected Total | 15 | 8935.9 |  |  |  |
|  | FS | Model | 3 | 12077 | 4025.8 | 17.8 | 0.0001 |
|  |  | Error | 12 | 2711.4 | 226.0 |  |  |
|  |  | Corrected Total | 15 | 14788 |  |  |  |
|  |  |  |  |  |  |  |  |
| Fine C | CT | Model | 3 | 56.7 | 18.9 | 11.5 | 0.0008 |
|  |  | Error |  | 19.7 | 1.64 |  |  |
|  |  | Corrected Total | 15 | 76.4 |  |  |  |
|  | NT | Model | 3 | 351.5 | 117.2 | 11.95 | 0.0006 |
|  |  | Error | 12 | 117.7 | 9.80 |  |  |
|  |  | Corrected Total | 15 | 469.1 |  |  |  |
|  | FS | Model | 3 | 426.0 | 142.0 | 4.92 | 0.019 |
|  |  | Error | 12 | 346.0 | 28.84 |  |  |
|  |  | Corrected Total | 15 | 772.1 |  |  |  |
|  |  |  |  |  |  |  |  |
| POC | CT | Model | 2 | 89.6 | 44.8 | 15.7 | 0.0012 |
|  |  | Error | 9 | 25.7 | 2.86 |  |  |
|  |  | Corrected Total | 11 | 115.3 |  |  |  |
|  | NT | Model | 2 | 856.0 | 428.0 | 4.42 | 0.046 |
|  |  | Error | 9 | 871.4 | 96.8 |  |  |
|  |  | Corrected Total | 11 | 1727.4 |  |  |  |
|  | FS | Model | 2 | 247.0 | 123.5 | 1.08 | 0.379 |
|  |  | Error | 9 | 1026.2 | 114.0 |  |  |
|  |  | Corrected Total | 11 | 1273.2 |  |  |  |
