# Supplementary material for: Soil Aggregates and Associated Organic Matter under Conventional Tillage, No-Tillage, and Forest Succession after Three Decades
Source: PLoS One. 2014 Jan 20;9(1):e84988. doi: 10.1371/journal.pone.0084988 (PMC3896348; doi:10.1371/journal.pone.0084988)
Supplement: Table S4 — 1. ANOVA results for Figure 4 in soil depth 5–15 cm for land use. ANOVA table reports tests of significance among land uses (CT, NT, and FS) within a carbon fraction and size class. 2. ANOVA results for Figure 4 in soil depth 5–15 cm for aggregate size class. ANOVA table reports tests of significance among size classes (2000, 250–2000, 53–250 and <53 µm) within a carbon fraction and land use. (DOCX) [file pone.0084988.s004.docx]

Table S4-1. ANOVA results for Figure 4 in soil depth 5-15 cm for land use. ANOVA table reports tests of significance among land uses (CT, NT, and FS) within a carbon fraction and size class.

| *C fraction* | *Size* | *Source* | *DF* | *SS* | *M1* | *F* | *Pr>F* |
| --- | --- | --- | --- | --- | --- | --- | --- |
| SOC | >2000 | Model | 2 | 138.8 | 69.4 | 4.78 | 0.039 |
|  |  | Error | 9 | 130.7 | 14.5 |  |  |
|  |  | Corrected Total | 11 | 269.4 |  |  |  |
|  | 250-2000 | Model | 2 | 236.2 | 118.1 | 4.77 | 0.0388 |
|  |  | Error | 9 | 223.1 | 24.7 |  |  |
|  |  | Corrected Total | 11 | 459.3 |  |  |  |
|  | 53-250 | Model | 2 | 137.9 | 69.0 | 3.90 | 0.0603 |
|  |  | Error | 9 | 159.2 | 17.7 |  |  |
|  |  | Corrected Total | 11 | 297.2 |  |  |  |
|  | <53 | Model | 2 | 10.4 | 5.19 | 0.46 | 0.648 |
|  |  | Error | 9 | 102.4 | 11.4 |  |  |
|  |  | Corrected Total | 11 | 112.8 |  |  |  |
|  |  |  |  |  |  |  |  |
| Fine C | >2000 | Model | 2 | 4.07 | 2.03 | 0.53 | 0.610 |
|  |  | Error | 9 | 34.86 | 3.87 |  |  |
|  |  | Corrected Total | 11 | 38.9. |  |  |  |
|  | 250-2000 | Model | 2 | 18.5 | 9.24 | 1.75 | 0.229 |
|  |  | Error | 9 | 47.6 | 5.29 |  |  |
|  |  | Corrected Total | 11 | 66.1 |  |  |  |
|  | 53-250 | Model | 2 | 9.04 | 4.52 | 0.88 | 0.449 |
|  |  | Error | 9 | 46.4 | 5.16 |  |  |
|  |  | Corrected Total | 11 | 55.5 |  |  |  |
|  | <53 | Model | 2 | 10.4 | 5.19 | 0.46 | 0.648 |
|  |  | Error | 9 | 102.4 | 11.4 |  |  |
|  |  | Corrected Total | 11 | 112.8 |  |  |  |
|  |  |  |  |  |  |  |  |
| POC | >2000 | Model | 2 | 30.5 | 15.3 | 4.53 | 0.0435 |
|  |  | Error | 9 | 30.3 | 3.36 |  |  |
|  |  | Corrected Total | 11 | 60.8 |  |  |  |
|  | 250-2000 | Model | 2 | 72.4 | 36.2 | 18.1 | 0.0007 |
|  |  | Error | 9 | 18.0 | 2.00 |  |  |
|  |  | Corrected Total | 11 | 90.4 |  |  |  |
|  | 53-250 | Model | 2 | 15.5 | 7.73 | 5.5 | 0.027 |
|  |  | Error | 9 | 12.6 | 1.39 |  |  |
|  |  | Corrected Total | 11 | 28.0 |  |  |  |
|  | <53 | Model |  |  |  |  |  |
|  |  | Error |  |  |  |  |  |
|  |  | Corrected Total |  |  |  |  |  |

Table S4-2. ANOVA results for Figure 4 in soil depth 5-15 cm for aggregate size class. ANOVA table reports tests of significance among size classes (2000, 250-2000, 53-250 and <53µm) within a carbon fraction and land use.

| *C fraction* | *Land Use* | *Source* | *DF* | *SS* | *M1* | *F* | *Pr>F* |
| --- | --- | --- | --- | --- | --- | --- | --- |
| SOC | CT | Model | 3 | 244.4 | 81.5 | 5.84 | 0.011 |
|  |  | Error | 12 | 167.4 | 13.9 |  |  |
|  |  | Corrected Total | 15 | 411.8 |  |  |  |
|  | NT | Model | 3 | 271.0 | 90.35 | 21.07 | <0.0001 |
|  |  | Error | 12 | 51.47 | 4.29 |  |  |
|  |  | Corrected Total | 15 | 322.5 |  |  |  |
|  | FS | Model | 3 | 733.6 | 244.5 | 7.40 | 0.005 |
|  |  | Error | 12 | 396.4 | 33.04 |  |  |
|  |  | Corrected Total | 15 | 1130 |  |  |  |
|  |  |  |  |  |  |  |  |
| Fine C | CT | Model | 3 | 12.38 | 4.13 | 0.85 | 0.494 |
|  |  | Error | 12 | 58.43 | 4.87 |  |  |
|  |  | Corrected Total | 15 | 70.81 |  |  |  |
|  | NT | Model | 3 | 22.98 | 7.66 | 2.89 | 0.079 |
|  |  | Error | 12 | 31.75 | 2.65 |  |  |
|  |  | Corrected Total | 15 | 54.72 |  |  |  |
|  | FS | Model | 3 | 36.77 | 12.26 | 1.04 | 0.409 |
|  |  | Error | 12 | 141.1 | 11.75 |  |  |
|  |  | Corrected Total | 15 | 177.8 |  |  |  |
|  |  |  |  |  |  |  |  |
| POC | CT | Model | 2 | 7.17 | 3.58 | 4.53 | 0.044 |
|  |  | Error | 9 | 7.11 | 0.79 |  |  |
|  |  | Corrected Total | 11 | 14.3 |  |  |  |
|  | NT | Model | 2 | 5.27 | 2.64 | 4.96 | 0.035 |
|  |  | Error | 9 | 4.78 | 0.53 |  |  |
|  |  | Corrected Total | 11 | 10.06 |  |  |  |
|  | FS | Model | 2 | 25.59 | 12.80 | 2.35 | 0.151 |
|  |  | Error | 9 | 48.94 | 5.44 |  |  |
|  |  | Corrected Total | 11 | 74.53 |  |  |  |
