# Supplementary material for: Soil Aggregates and Associated Organic Matter under Conventional Tillage, No-Tillage, and Forest Succession after Three Decades
Source: PLoS One. 2014 Jan 20;9(1):e84988. doi: 10.1371/journal.pone.0084988 (PMC3896348; doi:10.1371/journal.pone.0084988)
Supplement: Table S5 — 1. ANOVA results for Figure 5 in soil depth 15–28 cm for land use. ANOVA table reports tests of significance among land uses (CT, NT, and FS) within a carbon fraction and size class. 2. ANOVA results for Figure 5 in soil depth 15–28 cm for aggregate size class. ANOVA table reports tests of significance among size classes (2000, 250–2000, 53–250 and <53 µm) within a carbon fraction and land use. (DOCX) [file pone.0084988.s005.docx]

Table S5-1. ANOVA results for Figure 5 in soil depth 15-28 cm for land use. ANOVA table reports tests of significance among land uses (CT, NT, and FS) within a carbon fraction and size class.

| *Land use* | *Size* | *Source* | *DF* | *SS* | *M1* | *F* | *Pr>F* |
| --- | --- | --- | --- | --- | --- | --- | --- |
| SOC | >2000 | Model | 2 | 32.8 | 16.4 | 1.51 | 0.271 |
|  |  | Error | 9 | 97.6 | 10.8 |  |  |
|  |  | Corrected Total | 11 | 130.5 |  |  |  |
|  | 250-2000 | Model | 2 | 1.00 | 0.50 | 0.10 | 0.904 |
|  |  | Error | 9 | 44.1 | 4.90 |  |  |
|  |  | Corrected Total | 11 | 45.1 |  |  |  |
|  | 53-250 | Model | 2 | 5.22 | 2.61 | 0.73 | 0.509 |
|  |  | Error | 9 | 32.3 | 3.58 |  |  |
|  |  | Corrected Total | 11 | 37.5 |  |  |  |
|  | <53 | Model | 2 | 0.47 | 0.23 | 0.16 | 0.854 |
|  |  | Error | 9 | 13.1 | 1.46 |  |  |
|  |  | Corrected Total | 11 | 13.6 |  |  |  |
|  |  |  |  |  |  |  |  |
| Fine C | >2000 | Model | 2 | 22.4 | 11.2 | 2.15 | 0.172 |
|  |  | Error | 9 | 47.0 | 5.22 |  |  |
|  |  | Corrected Total | 11 | 69.4 |  |  |  |
|  | 250-2000 | Model | 2 | 0.074 | 0.04 | 0.01 | 0.99 |
|  |  | Error | 9 | 33.5 | 3.72 |  |  |
|  |  | Corrected Total | 11 | 33.5 |  |  |  |
|  | 53-250 | Model | 2 | 2.12 | 1.06 | 0.49 | 0.629 |
|  |  | Error | 9 | 19.6 | 2.17 |  |  |
|  |  | Corrected Total | 11 | 21.7 |  |  |  |
|  | <53 | Model | 2 | 0.47 | 0.23 | 0.16 | 0.854 |
|  |  | Error | 9 | 13.1 | 1.46 |  |  |
|  |  | Corrected Total | 11 | 13.6 |  |  |  |
|  |  |  |  |  |  |  |  |
| POC | >2000 | Model | 2 | 4.05 | 2.02 | 1.79 | 0.222 |
|  |  | Error | 9 | 10.2 | 1.13 |  |  |
|  |  | Corrected Total | 11 | 14.2 |  |  |  |
|  | 250-2000 | Model | 2 | 0.52 | 0.26 | 1.24 | 0.334 |
|  |  | Error | 9 | 1.89 | 0.21 |  |  |
|  |  | Corrected Total | 11 | 2.41 |  |  |  |
|  | 53-250 | Model | 2 | 0.52 | 0.26 | 2.40 | 0.146 |
|  |  | Error | 9 | 0.98 | 0.11 |  |  |
|  |  | Corrected Total | 11 | 1.51 |  |  |  |
|  | <53 | Model |  |  |  |  |  |
|  |  | Error |  |  |  |  |  |
|  |  | Corrected Total |  |  |  |  |  |

Table S5-2. ANOVA results for Figure 5 in soil depth 15-28 cm for aggregate size class. ANOVA table reports tests of significance among size classes (2000, 250-2000, 53-250 and <53µm) within a carbon fraction and land use.

| *C fraction* | *Land Use* | *Source* | *DF* | *SS* | *M1* | *F* | *Pr>F* |
| --- | --- | --- | --- | --- | --- | --- | --- |
| SOC | CT | Model | 3 | 248.3 | 82.77 | 8.80 | 0.002 |
|  |  | Error | 12 | 112.9 | 9.408 |  |  |
|  |  | Corrected Total | 15 | 361.2 |  |  |  |
|  | NT | Model | 3 | 101.1 | 33.69 | 9.68 | 0.0016 |
|  |  | Error | 12 | 41.74 | 3.48 |  |  |
|  |  | Corrected Total | 15 | 142.8 |  |  |  |
|  | FS | Model | 3 | 77.73 | 25.91 | 9.56 | 0.0017 |
|  |  | Error | 12 | 32.51 | 2.71 |  |  |
|  |  | Corrected Total | 15 | 110.24 |  |  |  |
|  |  |  |  |  |  |  |  |
| Fine C | CT | Model | 3 | 108.0 | 35.99 | 6.03 | 0.010 |
|  |  | Error | 12 | 71.6 | 5.97 |  |  |
|  |  | Corrected Total | 15 | 179.6 |  |  |  |
|  | NT | Model | 3 | 28.88 | 9.63 | 4.06 | 0.033 |
|  |  | Error | 12 | 28.43 | 2.37 |  |  |
|  |  | Corrected Total | 15 | 57.32 |  |  |  |
|  | FS | Model | 3 | 18.92 | 6.31 | 5.78 | 0.0111 |
|  |  | Error | 12 | 13.10 | 1.09 |  |  |
|  |  | Corrected Total | 15 | 32.02 |  |  |  |
|  |  |  |  |  |  |  |  |
| POC | CT | Model | 2 | 1.031 | 0.516 | 3.67 | 0.068 |
|  |  | Error | 9 | 1.265 | 0.141 |  |  |
|  |  | Corrected Total | 11 | 2.296 |  |  |  |
|  | NT | Model | 2 | 0.171 | 0.085 | 0.39 | 0.685 |
|  |  | Error | 9 | 1.948 | 0.216 |  |  |
|  |  | Corrected Total | 11 | 2.119 |  |  |  |
|  | FS | Model | 2 | 5.81 | 2.90 | 2.66 | 0.1238 |
|  |  | Error | 9 | 9.83 | 1.09 |  |  |
|  |  | Corrected Total | 11 | 15.63 |  |  |  |
