# Supplementary material for: Soil Aggregates and Associated Organic Matter under Conventional Tillage, No-Tillage, and Forest Succession after Three Decades
Source: PLoS One. 2014 Jan 20;9(1):e84988. doi: 10.1371/journal.pone.0084988 (PMC3896348; doi:10.1371/journal.pone.0084988)
Supplement: Table S6 — ANOVA results for Figure 6. ANOVA table reports tests of significance among Land Uses (conventional tillage, no tillage, forest succession) by soil depth (0–5, 5–15, 15–28 cm) for the sum of C contents in all aggregate fractions. (DOCX) [file pone.0084988.s006.docx]

Table S6: ANOVA results for Figure 6. ANOVA table reports tests of significance among Land Uses (conventional tillage, no tillage, forest succession) by soil depth (0-5, 5-15, 15-28 cm) for the sum of C contents in all aggregate fractions.

| *Depth* | *Source* | *DF* | *SS* | *M1* | *F* | *Pr>F* |
| --- | --- | --- | --- | --- | --- | --- |
| 0-5 | Model | 2 | 543.6 | 271.8 | 27.1 | 0.0002 |
|  | Error | 9 | 90.19 | 10.02 |  |  |
|  | Corrected Total | 11 | 633.8 |  |  |  |
| 5-15 | Model | 2 | 11.71 | 5.85 | 7.77 | 0.0109 |
|  | Error | 9 | 6.779 | 0.75 |  |  |
|  | Corrected Total | 11 | 18.49 |  |  |  |
| 15-28 | Model | 2 | 0.071 | 0.035 | 0.09 | 0.912 |
|  | Error | 9 | 3.397 | 0.377 |  |  |
|  | Corrected Total | 11 | 3.468 |  |  |  |
