# Supplementary material for: Soil Aggregates and Associated Organic Matter under Conventional Tillage, No-Tillage, and Forest Succession after Three Decades
Source: PLoS One. 2014 Jan 20;9(1):e84988. doi: 10.1371/journal.pone.0084988 (PMC3896348; doi:10.1371/journal.pone.0084988)
Supplement: Table S7 — ANOVA results for Table 2. ANOVA table reports tests of significance among Land Uses (conventional tillage, no tillage, forest succession) by aggregate attribute (Dry mean weighted diameter, Wet mean weighted diameter, Aggregate Stability {wet/dry}) and soil depth (0–5, 5–15 cm). (DOCX) [file pone.0084988.s007.docx]

Table S7: ANOVA results for Table 2. ANOVA table reports tests of significance among Land Uses (conventional tillage, no tillage, forest succession) by aggregate attribute (Dry mean-weighted diameter, Wet mean-weighted diameter, Aggregate Stability {wet/dry}) and soil depth (0-5, 5-15 cm).

| *Agg Attribute* | *Depth* | *Source* | *DF* | *SS* | *M1* | *F* | *Pr>F* |
| --- | --- | --- | --- | --- | --- | --- | --- |
| Dry MWD | 0-5 | Model | 2 | 0.755 | 0.377 | 3.31 | 0.0838 |
|  |  | Error | 9 | 1.027 | 0.114 |  |  |
|  |  | Corrected Total | 11 | 1.781 |  |  |  |
|  | 5-15 | Model | 2 | 3.820 | 1.910 | 5.81 | 0.024 |
|  |  | Error | 9 | 2.958 | 0.329 |  |  |
|  |  | Corrected Total | 11 | 6.778 |  |  |  |
|  |  |  |  |  |  |  |  |
| Wet MWD | 0-5 | Model | 2 | 5.593 | 2.796 | 40.30 | <0.0001 |
|  |  | Error | 9 | 0.625 | 0.069 |  |  |
|  |  | Corrected Total | 11 | 6.217 |  |  |  |
|  | 5-15 | Model | 2 | 2.783 | 1.392 | 25.5 | 0.0002 |
|  |  | Error | 9 | 0.491 | 0.055 |  |  |
|  |  | Corrected Total | 11 | 3.274 |  |  |  |
|  |  |  |  |  |  |  |  |
| Aggregate | 0-5 | Model | 2 | 0.463 | 0.231 | 50.6 | <0.0001 |
| Stability |  | Error | 9 | 0.041 | 0.005 |  |  |
|  |  | Corrected Total | 11 | 0.504 |  |  |  |
|  | 5-15 | Model | 2 | 0.345 | 0.173 | 26.4 | 0.0002 |
|  |  | Error | 9 | 0.032 | 0.004 |  |  |
|  |  | Corrected Total | 11 | 0.093 |  |  |  |
