# Supplementary material for: Soil Aggregates and Associated Organic Matter under Conventional Tillage, No-Tillage, and Forest Succession after Three Decades
Source: PLoS One. 2014 Jan 20;9(1):e84988. doi: 10.1371/journal.pone.0084988 (PMC3896348; doi:10.1371/journal.pone.0084988)
Supplement: Table S8 — ANOVA results for Table 3. ANOVA table reports tests of significance among Land Uses (conventional tillage, no tillage, forest succession) by soil carbon component (soil organic carbon, particulate organic carbon, fine carbon, microbial biomass carbon) and soil depth (0–5, 5–15 cm). (DOCX) [file pone.0084988.s008.docx]

Table S8: ANOVA results for Table 3. ANOVA table reports tests of significance among Land Uses (conventional tillage, no tillage, forest succession) by soil carbon component (soil organic carbon, particulate organic carbon, fine carbon, microbial biomass carbon) and soil depth (0-5, 5-15 cm).

| *C fraction* | *Depth* | *Source* | *DF* | *SS* | *M1* | *F* | *Pr>F* |
| --- | --- | --- | --- | --- | --- | --- | --- |
| SOC | 0-5 | Model | 2 | 10.47 | 5.232 | 5.30 | 0.0302 |
|  |  | Error | 9 | 8.892 | 0.988 |  |  |
|  |  | Corrected Total | 11 | 19.36 |  |  |  |
|  | 5-15 | Model | 2 | 0.054 | 0.027 | 0.16 | 0.854 |
|  |  | Error | 9 | 1.509 | 0.168 |  |  |
|  |  | Corrected Total | 11 | 1.563 |  |  |  |
|  |  |  |  |  |  |  |  |
| POC | 0-5 | Model | 2 | 5.593 | 2.796 | 40.30 | <0.0001 |
|  |  | Error | 9 | 0.625 | 0.069 |  |  |
|  |  | Corrected Total | 11 | 6.217 |  |  |  |
|  | 5-15 | Model | 2 | 5.662 | 2.831 | 20.78 | 0.0004 |
|  |  | Error | 9 | 1.226 | 0.136 |  |  |
|  |  | Corrected Total | 11 | 6.888 |  |  |  |
|  |  |  |  |  |  |  |  |
| Fine C | 0-5 | Model | 2 | 0.738 | 0.369 | 0.75 | 0.501 |
|  |  | Error | 9 | 4.444 | 0.494 |  |  |
|  |  | Corrected Total | 11 | 5.182 |  |  |  |
|  | 5-15 | Model | 2 | 0.030 | 0.015 | 0.12 | 0.892 |
|  |  | Error | 9 | 1.144 | 0.127 |  |  |
|  |  | Corrected Total | 11 | 1.174 |  |  |  |
|  |  |  |  |  |  |  |  |
| MBC | 0-5 | Model | 2 | 6390 | 3195 | 1.41 | 0.2929 |
|  |  | Error | 9 | 20371 | 2264 |  |  |
|  |  | Corrected Total | 11 | 26762 |  |  |  |
|  | 5-15 | Model | 2 | 4001 | 2001 | 5.62 | 0.0254 |
|  |  | Error | 9 | 3172 | 352 |  |  |
|  |  | Corrected Total | 11 | 7174 |  |  |  |
