# Supplementary material for: Soil Aggregates and Associated Organic Matter under Conventional Tillage, No-Tillage, and Forest Succession after Three Decades
Source: PLoS One. 2014 Jan 20;9(1):e84988. doi: 10.1371/journal.pone.0084988 (PMC3896348; doi:10.1371/journal.pone.0084988)
Supplement: Table S9 — ANOVA results for Table 4. ANOVA table reports tests of significance among Land Uses (conventional tillage, no tillage, forest succession) by aggregate size fraction (>2000, 250–2000, 53–250, and <53 µm) and soil depth (0–5, 5–15, 15–28 cm). (DOCX) [file pone.0084988.s009.docx]

Table S9: ANOVA results for Table 4. ANOVA table reports tests of significance among Land Uses (conventional tillage, no tillage, forest succession) by aggregate size fraction (>2000, 250-2000, 53-250, and <53 µm) and soil depth (0-5, 5-15, 15-28 cm).

| *Agg Size* | *Depth* | *Source* | *DF* | *SS* | *M1* | *F* | *Pr>F* |
| --- | --- | --- | --- | --- | --- | --- | --- |
| >2000 | 0-5 | Model | 2 | 458.0 | 229.0 | 9.39 | 0.0063 |
|  |  | Error | 9 | 219.4 | 24.4 |  |  |
|  |  | Corrected Total | 11 | 677.4 |  |  |  |
|  | 5-15 | Model | 2 | 154.6 | 77.3 | 4.36 | 0.0474 |
|  |  | Error | 9 | 159.6 | 17.7 |  |  |
|  |  | Corrected Total | 11 | 314.3 |  |  |  |
|  | 15-28 | Model | 2 | 321.3 | 160.6 | 2.54 | 0.1333 |
|  |  | Error | 9 | 568.7 | 63.2 |  |  |
|  |  | Corrected Total | 11 | 889.9 |  |  |  |
|  |  |  |  |  |  |  |  |
| 250-2000 | 0-5 | Model | 2 | 237.5 | 118.7 | 3.53 | 0.0738 |
|  |  | Error | 9 | 302.8 | 33.6 |  |  |
|  |  | Corrected Total | 11 | 540.3 |  |  |  |
|  | 5-15 | Model | 2 | 302.6 | 151.3 | 13.94 | 0.0018 |
|  |  | Error | 9 | 97.7 | 10.9 |  |  |
|  |  | Corrected Total | 11 | 400.4 |  |  |  |
|  | 15-28 | Model | 2 | 37.8 | 13.9 | 1.90 | 0.2049 |
|  |  | Error | 9 | 95.7 | 7.30 |  |  |
|  |  | Corrected Total | 11 | 93.4 |  |  |  |
|  |  |  |  |  |  |  |  |
| 53-250 | 0-5 | Model | 2 | 372.3 | 186.2 | 3.73 | 0.0661 |
|  |  | Error | 9 | 449.1 | 49.9 |  |  |
|  |  | Corrected Total | 11 | 821.4 |  |  |  |
|  | 5-15 | Model | 2 | 35.4 | 17.7 | 5.11 | 0.0329 |
|  |  | Error | 9 | 31.2 | 3.5 |  |  |
|  |  | Corrected Total | 11 | 66.6 |  |  |  |
|  | 15-28 | Model | 2 | 27.6 | 13.8 | 3.66 | 0.0688 |
|  |  | Error | 9 | 34.0 | 3.77 |  |  |
|  |  | Corrected Total | 11 | 61.6 |  |  |  |
|  |  |  |  |  |  |  |  |
| Whole | 0-5 | Model | 2 | 103.8 | 51.9 | 23.3 | 0.0003 |
| Soil |  | Error | 9 | 20.0 | 2.22 |  |  |
|  |  | Corrected Total | 11 | 123.8 |  |  |  |
|  | 5-15 | Model | 2 | 2.77 | 1.39 | 9.07 | 0.007 |
|  |  | Error | 9 | 1.38 | 0.15 |  |  |
|  |  | Corrected Total | 11 | 4.14 |  |  |  |
|  | 15-28 | Model | 2 | 0.193 | 0.097 | 2.61 | 0.128 |
|  |  | Error | 9 | 0.334 | 0.037 |  |  |
|  |  | Corrected Total | 11 | 0.527 |  |  |  |
